# Lay Counselor Delivery of Trauma-Focused Cognitive Behavioral Therapy (TF-CBT): A Systematic Review

**DOI:** 10.1007/s40653-026-00852-z

**Published:** 2026-03-26

**Authors:** Laura B. Godfrey, Nevita George, Amy Hyoeun Lee

**Affiliations:** Hofstra University, Hempstead, United States

**Keywords:** TF-CBT, Lay counselors, Implementation, Modification, Systematic review

## Abstract

Lay counselor delivery of trauma-focused cognitive behavioral therapy (TF-CBT) may be a promising strategy to increase access to trauma-focused care and prevent trauma-related health disparities among youth in low-resource settings in the United States. This pre-registered systematic review sought to synthesize *how* TF-CBT has been tailored for lay counselor delivery globally, which can inform broader dissemination. Specifically, we examined (1) the modification process, (2) treatment modifications, and (3) implementation strategies (e.g., training and supervision) to support lay counselor delivery of TF-CBT. In May 2024, a literature search was conducted across PsycINFO, PubMed, MEDLINE, and PILOTS. Grey literature was identified through Google Scholar and ProQuest. A narrative synthesis summarized findings by research question. Ten trials (21 articles) met inclusion criteria and described modifications or implementation strategies for lay counselor-delivered TF-CBT. All trials implemented culturally tailored versions of TF-CBT, with most (90%) conducted in low- and middle-income countries. The modification process relied on input from local experts through community-researcher collaborations. TF-CBT modifications were contextual (e.g., modality) or surface-level (e.g., language), with no trials reporting modifications to core components. Implementation strategies included enhancing training and supervision, addressing implementation barriers, and preserving counselor wellbeing. Findings suggest that TF-CBT can be successfully delivered by lay counselors with peripheral modifications, enhanced counselor supports, and strong community partnerships. This review provides a foundation for future research aimed at disseminating lay counselor-delivered TF-CBT in low-resource settings – a critical direction to close the mental health treatment gap and promote health equity in the United States and globally.

**Supplementary Information:**

The online version contains supplementary material available at https://doi.org/10.1007/s40653-026-00852-z.

An estimated 60–80% of youth in the United States (US) have experienced a traumatic event (McLaughlin et al., 2013; Turner et al., 2010). Trauma exposure (i.e., an event involving actual or threatened death, serious injury, or sexual violence; American Psychiatric Association, 2013) is linked to a range of mental health consequences, including posttraumatic stress disorder (PTSD; McLaughlin et al., 2013), and associated with impairments in cognitive, academic, social, and emotional functioning (De Bellis et al., 2009; Trickett et al., 2011). Effective evidence-based treatments for childhood trauma sequelae include Trauma-Focused Cognitive Behavioral Therapy (TF-CBT; Cohen et al., 2017), an evidence-based mental health treatment for youth with trauma-related mental health needs (Cohen et al., 2017). TF-CBT is the most widely studied youth PTSD treatment and deemed a *well-established* treatment with robust effectiveness in reducing symptoms of posttraumatic stress, depression, anxiety, and traumatic grief in children and adolescents ages 3–18 (Dorsey et al., 2017; Silverman et al., 2008; Thielemann et al., 2022). However, most youth (up to 80%) in need of mental health services in the US do not receive care or evidence-based interventions such as TF-CBT (Kataoka et al., 2002). This treatment gap affects health and wellbeing outcomes throughout the lifespan, which contribute to leading causes of death and disability and drive racial and socioeconomic health disparities (Felitti et al., 1998; López et al., 2017). Strategies to improve access to evidence-based treatments for childhood trauma-related mental health needs such as TF-CBT are urgently needed to improve public health and long-term outcomes for youth in the US.

The shortage of mental health providers is one of the most potent barriers in access to evidence-based mental health services. National data suggest that the existing workforce in the US holds capacity to meet less than 30% of mental health needs (US Bureau of Health Workforce, 2024), and that four-fifths of US counties are partial or whole Mental Health Professional Shortage areas (Cummings et al., 2013). These workforce shortages are most pronounced in low-resource, community-based settings (Cook et al., 2013; Dinwiddie et al., 2013; Mongelli et al., 2020). As a result, the mental health treatment gap disproportionately affects low-income and racially minoritized youth, who face higher rates of trauma exposure (Andrews et al., 2015; Hatch & Dohrenwend, 2007) yet have lower rates of access to services (Cummings et al., 2013; Merikangas et al., 2011). These data suggest that the pervasive mental health treatment gap and trauma-related health disparities cannot be fully remediated without solutions for the mental health workforce shortage in under-resourced settings.

One strategy to address the mental health workforce shortage and expand access to mental health services is *task-shifting*. Task-shifting involves training lay counselors (i.e., persons with little to no formal mental health training) such as teachers, community health workers, volunteers, or people with lived experience, to deliver interventions that are typically delivered by mental health professionals (Javadi et al., 2017; World Health Organization, 2007). A robust body of research, including four previous reviews, has demonstrated that mental health treatments delivered by lay counselors are effective in improving clinical outcomes (Barnett et al. 2018a, b; Connolly et al. 2021; Singla et al. 2017; van Ginneken et al. 2013). For instance, a systematic review and meta-analysis of 1072 studies in low- and middle-income countries (LMICs) by Connolly and colleagues (2021) found a significant, medium-sized effect for lay counselor delivered interventions. In their review of 39 trials testing community health worker delivery of evidence-based mental health interventions, ten of which tested trauma-focused interventions, Barnett and colleagues (2018) found that lay counselor-delivered treatments led to symptom reduction. Currently, most empirical support for lay counselor-delivered mental health treatments comes from implementation efforts in LMICs (Barnett et al. 2018a, b; Connolly et al. 2021). Nonetheless, this strategy holds promise for addressing workforce shortages in the US, a key driver of mental health disparities. Lay counselor-delivery may also be responsive to unique barriers faced by minoritized communities that exacerbate disparities in service utilization, including mistrust of medical systems (LaVeist et al., 2009) and stigma in seeking mental health services (Cook et al., 2017; Gary, 2005). Given the effectiveness and implementation advantages of task-shifting, lay counselor delivery of trauma-focused treatments is a promising strategy for closing treatment gaps and promoting health equity both internationally and domestically.

Effective dissemination of task-shifting models requires an understanding of the strategies needed to support their implementation (Padmanathan & De Silva, 2013). As such, scholars have called for research that moves beyond demonstrating effectiveness towards identifying intervention modifications and implementation strategies for lay counselors (Barnett et al. 2018a, b; Barnett, Lau et al., 2018; Singla et al., 2017). Implementation science proposes that modification of evidence-based interventions may be necessary for successful translation of research to practice. Modification is the process of altering the design or delivery of an intervention to improve its fit in a given context (Wiltsey Stirman et al., 2019). Modification may include alterations to the intervention itself and/or alterations to implementation strategies. Implementation strategies are broadly defined as actions used to enhance adoption, implementation, and sustainability of evidence-based interventions (Powell et al., 2015; Proctor et al., 2013), such as clinician training and supervision and the process of building organizational buy-in. Despite their importance in bridging the treatment gap, modifications are not consistently documented in the literature, limiting the uptake of interventions in real-world settings (Wiltsey Stirman et al., 2019).

The Framework for Reporting Adaptations and Modifications—Expanded (FRAME; Wiltsey Stirman et al., 2019) is a guide developed to systematize the reporting of modifications. According to FRAME, modifications may be made to intervention content (aspects of treatment), context (how the treatment is delivered), training, and other implementation and scale-up strategies. Although modification may take many forms, including tailoring treatment setting and format, shortening or lengthening treatment, adding elements, and changing the packaging of treatment, most literature describing modifications to evidence-based treatments has focused on cultural adaptation. For instance, a relevant systematic review by Lange and colleagues (2022) characterized adaptations to youth trauma-focused interventions and found that most adaptations were made to address cultural factors. Although the authors reported lay counselor treatment delivery as a modification in some studies, further details of how interventions were tailored for lay counselor delivery was outside of the scope of the review (Lange et al., 2022). Modifications improve feasibility, acceptability, and clinical effectiveness of interventions (Wiltsey Stirman et al., 2019), yet little is known about which modifications are used for task-shifting. For instance, two systematic reviews examining the effectiveness of lay counselor-delivered treatments found that training, supervision, and other supports were inconsistently reported (Barnett et al. 2018a, b; Singla et al. 2017). No reviews have characterized how to support lay counselor-delivered treatments, which significantly limits scale-up of task-shifting.

Although TF-CBT was developed to be delivered by trained mental health professionals, task-shifting presents an important opportunity to expand access to this highly effective treatment and prevent the long-term consequences of childhood trauma. Global mental health researchers have implemented lay counselor-delivered TF-CBT in LMICs, where workforce shortages and youth trauma-related mental health needs are particularly pronounced. For instance, trials of lay counselor-delivered TF-CBT have been included in prior reviews that demonstrate the effectiveness of TF-CBT (Thielemann et al., 2022), the effectiveness of task-shifting (Barnett et al. 2018a, b), and adaptations to trauma-focused treatments (Lange et al., 2022). However, no prior systematic reviews have focused on lay counselor-delivered TF-CBT specifically or synthesized the modifications and strategies used to support implementation of this intervention.

Taken together, lay counselor delivery of TF-CBT may be a promising avenue for interrupting the negative sequelae of youth trauma exposure, ameliorating the mental health treatment gap, and advancing public health. Existing reviews have demonstrated the effectiveness of lay counselor-delivered evidence-based treatments including TF-CBT, but none have attempted to synthesize strategies used to support implementation of task-shifting models. Understanding how TF-CBT is modified and which components are altered to yield successful delivery by lay counselors could be critical to advancing dissemination in low-resource settings. To fill this gap in the literature, this pre-registered systematic review [MASKED] sought to characterize how prior studies have modified and supported implementation of TF-CBT delivered by lay counselors. Specifically, we aimed to answer the following questions: (1) What is the process of identifying and operationalizing modifications to TF-CBT? (2) What intervention modifications are made? and (3) What implementation strategies (e.g., training and supervision) are used to support lay counselor delivery of TF-CBT?

## Materials and Methods

### Search Strategy

**Fig. 1:**
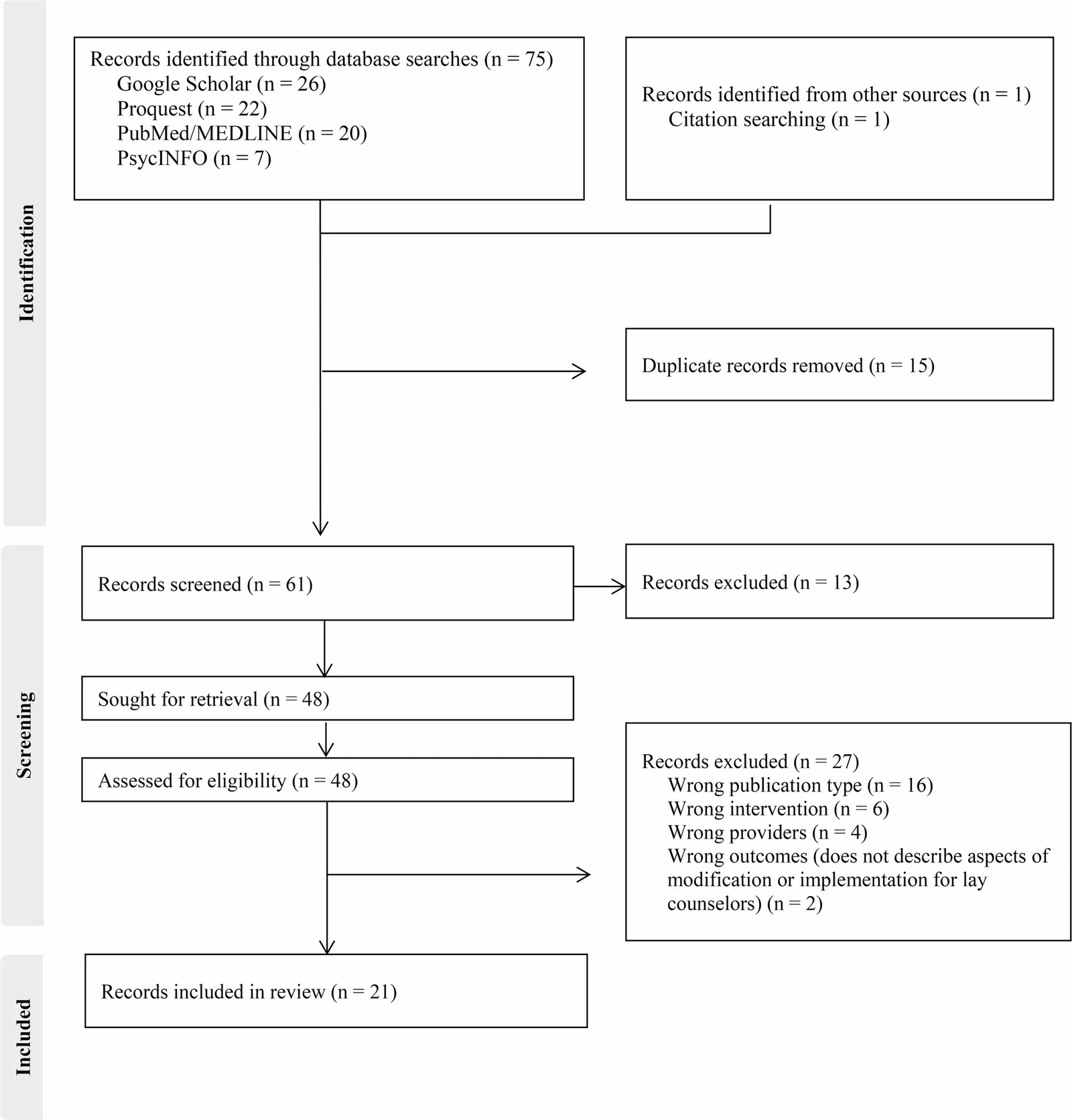
PRISMA flow diagram

We conducted a comprehensive literature search to identify studies describing modifications and implementation of lay counselor-delivered TF-CBT. On May 5th, 2024, the search was conducted across four databases – PsycINFO, PubMed, MEDLINE, and PILOTS, and two additional search engines – Google Scholar and ProQuest, to identify gray literature. Reference lists of included articles were also manually screened to identify additional relevant studies. A keyword-based Boolean search string (Table 1) was applied to each search engine. The search strategy included terms for (1) lay counselors, (2) modification/implementation, and (3) TF-CBT. To maximize sensitivity, we did not make database-specific syntax adjustments (e.g., controlled vocabulary terms or field tags) and terms were applied broadly across all searchable fields within each database using default search behavior. This approach minimized the risk of excluding relevant records due to differences in indexing across databases and variability in terminology describing lay counselors. The search was not limited by publication date, study design, or location, and was re-run prior to analyses (August 2024). All procedures were conducted in accordance with PRISMA guidelines (Fig. 1).

**Table 1:** Search string

(“lay worker” OR “lay health worker” OR LHW OR “lay mental health worker” OR “lay counselor” OR “lay health counselor” OR “lay counsellor” OR “community health worker” OR CHW OR “task shift*” OR “peer deliver*” OR “peer support” OR “nonprofessional” OR “community volunteer”)
AND
(Adapt* OR tailor* OR implement* OR “implementation strateg*” OR “implementation facilitation” OR optimiz* OR modif* OR train* OR supervis*) AND
(“trauma focused cognitive behavioral therapy” OR “trauma focused CBT” OR “TF CBT” OR “trauma focused cognitive behavioural therapy”)

The search string was applied to all databases (PubMed/MEDLINE, PsycInfo, PILOTS, Google Scholar, ProQuest)

Two independent coders (first and second authors) screened all titles, abstracts, and full texts of identified articles for relevance using Covidence (Veritas Health Innovation, 2024), an online software for systematic reviews. Discrepancies were resolved through consensus (i.e., reaching 100% agreement across coders) with consultation with the senior author as needed, therefore initial inter-rater reliability was not calculated. Record inclusion was contingent on the following criteria: (1) available in English; (2) intervention is TF-CBT; (3) TF-CBT is delivered, or intended to be delivered, by lay counselors (i.e., non-mental health professionals); and (4) includes description of the modification process, intervention modifications, and/or implementation strategies for lay counselor delivery. Records were excluded if they reported (1) outcomes of lay counselor-delivered TF-CBT but did not describe modification components; (2) modifications to psychotherapy interventions for lay counselors broadly but nonspecific to TF-CBT; and (3) only cultural modifications of lay counselor-delivered TF-CBT and not modifications that are relevant to task-shifting. Given our focus on modifications, empirical outcomes were not necessary for inclusion. This allowed us to identify components of modification that may have been excluded from prior reviews focused on characterizing treatment effectiveness.

### Data Extraction

A deductive coding approach was employed to analyze qualitative data given the efficacy in applying a pre-established theoretical framework within data analyses (Glaser & Strauss, 2017). Constructs for data extraction were informed by domains outlined by the Framework for Reporting Adaptations and Modifications—Expanded (FRAME; Wiltsey Stirman et al., 2019), a guide developed improve consistency in reporting of intervention modifications. A codebook with definitions of each variable was created. Variables included trial characteristics (e.g., study design and setting, patient population, lay counselor characteristics, TF-CBT delivery), and for our main research questions, text excerpts describing the modification process, training, supervision, and implementation strategies were extracted for later narrative synthesis. The data extraction form is presented in Supplementary Table 1. Two coders (first and second authors) independently extracted data from an initial set of three studies to ensure consensus and refine the codebook. Coders met to review discrepancies and achieve full consensus (100% agreement) before independently extracting data from the remaining studies. The coders met on a weekly basis to cross-verify data by comparing the extracted text excerpts. Discrepancies were resolved collaboratively through consensus with the involvement of the senior author as needed. All qualitative data were managed and analyzed using Microsoft Excel.

## Bias/Quality Assessment

Risk of bias and quality assessment were examined independently by two coders (first and second authors) for each full-text article included using the Quality Assessment for Diverse Studies (QuADS) tool (Harrison et al., 2021). QuADS was selected given its flexibility in assessing bias across methodology (quantitative, qualitative, mixed methods). QuADS contains 13 domains (e.g., “Theoretical or conceptual underpinning to the research”) rated on a scale of 0 (not addressed) to 3 (fully addressed) based on criteria outlined in the QuADS checklist tool (Harrison et al., 2021). Independent coders rated the included studies on each domain and discrepancies were resolve through consensus. Quality assessment scores for each study are presented in Supplementary Table 2. The most common reporting weaknesses were recruitment transparency (e.g., final sample size reported but not the total number of individuals approached) and stakeholder involvement. Although stakeholder collaboration was universal, it was often described in the context of implementation and fewer studies described the role of stakeholders in research question and study design selection. Strengths included tailoring data collection tools to the local setting, appropriate selection of study designs and data analytic methods, and thorough discussion of strengths and limitation. Because the aim of this review was to characterize reported strategies for implementation of lay counselor-delivered TF-CBT rather than evaluate effectiveness, quality assessment did not inform data synthesis, and we did not conduct additional risk of bias assessments specific to effectiveness trials. Therefore, the internal validity of effectiveness outcomes was not formally appraised, and conclusions related to effectiveness should be interpreted descriptively.

### Qualitative Coding: Narrative Synthesis

We applied a narrative synthesis approach to summarize findings given its strength in integrating and considering variations across qualitative and quantitative findings (Popay et al., 2006). Tabulation was used to understand trial characteristics (e.g., participants, lay counselors, version of TF-CBT, delivery setting). To answer the main research questions, two independent coders (first and second authors) conducted conceptual, content analyses to identify patterns of meaning and recurring themes within extracted text excerpts pertaining to the modification process, TF-CBT modifications, and implementation strategies (Drisko & Maschi, 2015). Coders reviewed extracted text and collaboratively generated textual descriptions to summarize this information per study and identify themes. Frequencies of reported modifications and strategies across studies were tabulated to highlight prevalence of specific modification and implementation activities, and textual descriptions are included to provide contextual depth (Popay et al., 2006).

## Results

Our search yielded 75 records and after removal of duplicates, 61 were assessed for eligibility. Of these, 21 records met criteria for inclusion (Fig. 1). Most were published in peer-reviewed journals (*n* = 20), and one dissertation was included. Excluded full-text articles and reasons are presented in Supplementary Table 3. The 21 records reflect a total of 10 larger trials. To avoid duplicate reporting, results are described by trial. Trials with multiple included articles are cited in-text by the record that contains the most detail pertaining to the parent trial. The full list of records per trial are presented in Tables 2 and 3. Information on trial characteristics including location, participants, and lay counselors are presented in Table 2 and summarized below. Findings are then presented by research question, reflecting (1) the modification process, (2) intervention modifications, and (3) implementation strategies (e.g., training and supervision) to support TF-CBT delivery by lay counselors.

**Table 2:** Characteristics of trials of lay counselor-delivered TF-CBT included in systematic review

	Trial Characteristics		Trial Participants			Lay Counselor Characteristics
Trial	Design	Country	*N*; Condition	Age, Gender	*N*; Role	Selection & Qualifications
1. Dorsey et al. (2019) Dorsey et al. (2020a, b) Dorsey et al., (2023) Johnson et al. (2024) Martin (2022) Meza et al. (2020) Triplett et al. (2021) Triplett et al. (2023a, b) Triplett et al. (2023a, b)	Stepped wedge cluster RCT^a^	Kenya	1280; TF-CBT in health or education sector	Ages 11–14 50% girls/boys	240; Teachers and community health volunteers.	Nominated by local leadership; Qualifications included: good with children, may have counseling experience (not required), have time to deliver the program, no plans for leaving the area.
2. Dorsey et al., (2022) Dorsey et al. (2020a, b) Woods-Jaeger et al. (2017)	RCT^a^	Tanzania & Kenya	640; TF-CBT (320), usual care (320)	Ages 7–13 50% girls/boys	12; NR	Hired by local organization; Expected to have experience/interest working with children/families. All had undergraduate degrees.
3. Murray et al. (2013a, b) Murray et al. (2014)	Feasibility study^b^	Zambia	40	Ages 4–18 100% girls (*N* = 21 treatment completers)	19; University students, staff from university, organizations, and hospitals.	Experience and education varied; 3 had some clinical training.
4. Li et al. (2023)	RCT	China	234; TF-CBT (118), TAU (116)	Ages 9–12	15; University students	Recruited from university; None had experience in mental health treatment.
5. Murray et al. (2013a, b)	Feasibility study^b^	Zambia	94	Ages 5–18 (*N* = 58 completers)	18; NR	Counselors with minimal formal training.
6. Murray et al. (2015)	RCT^b^	Zambia	257; TF-CBT (131), TAU (126)	Ages 5–18	20; NR	Backgrounds varied; All had high school education and basic communication/social skills.
7. O’Callaghan et al. (2013)	RCT	Democratic Republic of Congo	52; TF-CBT (24), waitlist (28)	Ages 12–17; 100% girls	NR; Nonclinical social workers	Staff from the local organization who provide psychosocial support to youth.
8. O’Callaghan et al. (2015)	RCT	Democratic Republic of Congo	50; TF-CBT (26, non-trauma focused (24)	Ages 8–17; 29 boys, 21 girls	One teacher with assistance from two nonclinical social workers	Teacher from prior trial and social workers from organization/funder.
9. O’Donnell et al. (2014)	Feasibility study^a^	Tanzania	64	Ages 7–13	4; NR	Hired by local organizations; Desired qualifications: experience working with children and/or counseling, bilingual in English, willingness to be trained and work with the US investigators. 3 had some university education, 3 had prior work with children, none had mental health treatment experience.
10. Wang et al. (2016)	RCT	Haiti	58; TF-CBT (38), control (20)	Ages 6–20; 68% girls (TF-CBT group)	24; community members from local churches and organizations, university students.	Must be part of a local organization that supports participation. Requirements varied based on role. Counselors from churches: leader endorsement, 6th grade education, some prior training, and ≥3 years working with local youth. Organization volunteers: bachelor’s degree and ≥1 year working with local youth. Students: ≥2 years of undergraduate work and some training.

*NR* not reported, *RCT* randomized controlled trial, *TF*-*CBT* trauma-focused cognitive behavioral therapy, *TAU* treatment as usual. Superscripts are used to indicate trials that were conducted by the same researcher-community partnerships over multiple research initiatives

**Table 3:** Intervention Characteristics across trials of lay counselor-delivered TF-CBT

	TF-CBT Delivery			Modifications		
Trial	TF-CBT	Delivery Setting	Format	Frequency & Duration	Context	Content
1. Dorsey et al. (2019) Dorsey et al. (2020a, b) Dorsey et al., (2023) Johnson et al. (2024) Martin (2022) Meza et al. (2020) Triplett et al. (2021) Triplett et al. (2023a, b) Triplett et al. (2023a, b)	Culturally tailored (Pamoja Tunaweza)	“Village clusters”: Schools and surrounding community	Group and individual	8 weekly 60 min sessions.	Setting, modality, number of sessions	NR
2. Dorsey et al., (2022) Dorsey et al. (2020a, b) Woods-Jaeger et al. (2017)	Culturally tailored (Pamoja Tunaweza)	NR	Group and individual	30–120 min sessions over 12 consecutive weeks.	Modality, duration	Language
3. Murray et al. (2013a, b) Murray et al. (2014)	Culturally tailored	NR	Individual	Weekly sessions (range 12–32).	NR	Language
4. Li et al. (2023)	Culturally tailored (Power up Children’s Psychological Immunity)	Schools	Group and individual	50 min sessions over 9 consecutive weeks (range 10–12)	Setting, modality	NR
5. Murray et al. (2013a, b)	Culturally tailored	Local centers (hospice, centers for street youth, childcare for HIV-affected youth, health clinic)	Individual	Weekly 30–120 min sessions over an average of 11 weeks (range 8–23).	Setting, duration	Language
6. Murray et al. (2015)	Culturally tailored	Local centers (home-based care, center for street youth, health clinic, school/residential)	Individual	Weekly 60–90 min sessions (range 10–16)	Setting	NR
7. O’Callaghan et al. (2013)	Culturally tailored	Hallway in local school	Group and individual	120 min/day, 3 days/week for 5 weeks (15 sessions).	Setting, modality, frequency	NR
8. O’Callaghan et al. (2015)	Culturally tailored	Under a tent in a field attached to a local school	Group	90 min sessions 3x/week (9 sessions)	Setting, modality, frequency	NR
9. O’Donnell et al. (2014)	Culturally tailored TF-CBT for Traumatic Grief	Community buildings	Group and individual	12 weekly 60 min sessions.	Setting, modality	NR
10. Wang et al. (2016)	Culturally tailored spiritually oriented TF-CBT	NR	Individual	12 weekly sessions. Some required up to 6 months and > 1 session/week.	Frequency	NR

*NR* not reported, *TF*-*CBT* trauma-focused cognitive behavioral therapy

### Trial Characteristics

Nine trials (90%) took place in LMICs (Zambia, The Democratic Republic of Congo, Tanzania, Kenya, and Haiti), one (10%) in an upper-middle-income country (China) (World Bank, 2025). All (100%) implemented a culturally tailored version of TF-CBT. The sample size of trial participants (i.e., TF-CBT recipients) ranged from 38 to 1280. Participant ages ranged from 4 years old to 20 years old. Two trials (20%) reported youth race/ethnicity (Li et al., 2023; Murray et al., 2015) and six (60%) reported their sex (Dorsey et al. 2020a, b; Murray et al. 2013a, b, 2015, p. 20; Murray et al. 2013a, b; O’Donnell et al. 2014; Wang et al. 2016). Nine trials (90%) reported effectiveness outcomes of lay counselor-delivered TF-CBT, all of which found that it improved clinical outcomes (Dorsey et al. 2020a, b; Li et al. 2023; Murray et al. 2013a, b, 2015; O’Callaghan et al. 2013, 2015; O’Donnell et al. 2014; Wang et al. 2016).

### Lay Counselor Characteristics

The number of lay counselors who delivered TF-CBT ranged from one to 240. Seven trials (70%) described the roles of the lay counselors in the community. Lay counselors were staff or volunteers from local communities and organizations, teachers, non-clinical social workers, and university students. Seven studies (70%) described how lay counselors were selected. Hiring was often facilitated through community partner organizations and qualifications were commonly described as general traits, such as interest or experience working with children, willingness and ability to deliver TF-CBT, and basic communication and social skills, rather than concrete criteria. Six trials (60%) reported the prior mental health training and education of lay counselors, which varied widely across and within studies given that these were not typically required qualifications. Only one trial reported that some prior mental health training was required (Wang et al., 2016). Education level of counselors ranged from completion of 6th grade to an undergraduate degree. Few studies reported additional demographic information of lay counselors. None reported counselor race or ethnicity, one reported age from a subset of counselors (Dorsey et al. 2020a, b) and two reported sex (Dorsey et al. 2020a, b; Murray et al. 2013a, b), both of which reported that most were female.

### What is the Process of Identifying and Operationalizing Modifications to TF-CBT for Delivery by Lay Counselors?

The modification process refers to ways in which the need for alterations (to TF-CBT and/or implementation strategies) was determined and operationalized, including who was involved and when modification took place relative to TF-CBT implementation. This process was inconsistently reported, and the level of detail and components of the process reported varied widely. Many described modifications as a multiphasic, ongoing process.

Most trials used a theoretical framework to guide this process, including the Apprenticeship Model of training and supervision (*n* = 5, 50%; Dorsey et al. 2020a, b; Dorsey et al. 2020a, b; Murray et al. 2013a, b, 2015) and the Design, Implementation, Monitoring, and Evaluation (DIME) model (*n* = 2, 20%; Murray et al. 2013a, b; Wang et al. 2016). The Apprenticeship Model underscores the role of lay counselors as experts in their local setting and embeds intervention tailoring as a key activity throughout implementation (Murray et al., 2011). In this model, potential modifications to TF-CBT are identified by lay counselors during training and throughout supervision and are reviewed by trainers, supervisors, and TF-CBT experts to ensure adherence to fidelity. The DIME model comprises a series of steps to facilitate successful program implementation that are anchored in researcher-community partnerships, including a qualitative local needs assessment and development and adaptation of assessment tools that precede modification and testing of the intervention in later steps (AMHR, 2000). In addition to these frameworks, one trial selected a stepped-wedge cluster randomized control trial design to embed an iterative, data-driven adaptation process within the same trial (Dorsey et al. 2020a, b). In this trial, TF-CBT is delivered over seven sequences. Findings from the first sequence elucidate modifiable implementation determinants that are collaboratively translated into an implementation facilitation plan by researchers and lay counselors to support TF-CBT delivery in subsequent sequences.

For some trials, the process of modification spanned multiple research initiatives within the same setting or researcher-community partnership (See **Table 2)**. For instance, three trials (Dorsey et al. 2020a, b; O’Donnell et al. 2014) reflect a series of projects conducted with the same longstanding community partners, wherein initial intervention modifications were tested in a feasibility study (O’Donnell et al., 2014), followed by an RCT testing intervention effectiveness (Dorsey et al. 2020a, b), and a larger RCT testing tailored implementation strategies that may support feasibility and sustainability of TF-CBT (Dorsey et al. 2020a, b). Similarly, (Murray et al. 2013a, b) tested modified TF-CBT in a feasibility study, which informed further evaluation of implementation in usual care (Murray et al. 2013a, b) and in a subsequent RCT (Murray et al., 2015). Another study implemented a tailored version of TF-CBT that was tested in the local setting in a prior study (Li et al., 2023).

A range of activities were used to identify and operationalize modifications pre-, during, and post-implementation. Pre-implementation, trials gathered preliminary recommendations and feedback on TF-CBT materials from local stakeholders (Dorsey et al. 2020a, b; Li et al. 2023; O’Donnell et al. 2014; Wang et al. 2016), which some accomplished through focus groups (Dorsey et al. 2020a, b; Li et al. 2023; O’Donnell et al. 2014). During implementation, modifications were identified based on feedback from lay counselors who were delivering TF-CBT. Feedback was gathered during training and supervision (Dorsey et al. 2020a, b; Murray et al. 2013a, b, 2015; O’Callaghan et al. 2013; Wang et al. 2016), interviews (Murray et al. 2013a, b), and during a mixed methods evaluation of intervention acceptability, feasibility, appropriateness, and implementation determinants (Dorsey et al. 2020a, b). One trial also developed a comprehensive database of the identified modifications (Murray et al. 2013a, b). Some trials described activities to identify modifications post-implementation, including gathering feedback from participants who refused treatment (Murray et al. 2013a, b), and feedback from counselors who continued delivering TF-CBT post-trial to identify modifications that they made to enhance sustainability in usual care (Dorsey et al. 2020a, b). Notably, all modification strategies involved engaging local personnel (e.g., lay counselors, staff from community organizations) as key informants given their role as experts of the local setting.

### What Intervention Modifications are Made to TF-CBT for Lay Counselor Delivery?

Intervention modifications refer to aspects of TF-CBT that were altered for lay counselor delivery, and are further characterized as context (i.e., how the intervention was delivered) or content modifications (i.e., intervention components) (Wiltsey Stirman et al., 2019). All trials reported modifications to TF-CBT for lay counselor delivery, and context modifications were more frequently reported (*n* = 9, 90%) than content modifications (*n* = 3, 30%). Additional detail on TF-CBT version, frequency, modality, and delivery setting is presented in Table 3.

#### Context Modifications

Nine trials (90%) reported context modifications, including alterations to the setting, modality, frequency, and/or duration of treatment. Originally, TF-CBT was developed for delivery through 12–16 weekly individual therapy sessions (50–90 min) with a licensed mental health clinician (Cohen et al., 2017). Of the seven trials (70%) that reported the setting of delivery, all implemented TF-CBT in non-specialty mental health settings, including schools, community organizations that provide other services to youth, and community spaces. In two trials, TF-CBT was implemented alongside usual services in the setting to facilitate sustainability, including 1-hour slots during the school day for lay counselors who were teachers (Dorsey et al. 2020a, b), and within programming in an organization that provides services to HIV-affected youth (Murray et al. 2013a, b). Six trials implemented group-delivered TF-CBT. In one trial, this was informed by focus group interviews with counselors prior to trial start (Dorsey et al. 2020a, b). Two studies used three-counselor “teams” to deliver TF-CBT (Dorsey et al. 2020a, b), where two counselors facilitated the child group, and one led the caregiver group. In terms of frequency, most trials (*n* = 8; 80%) implemented standard weekly sessions and two delivered three sessions per week. In one trial, the weekly frequency was modified during implementation. For some cases, multiple sessions were delivered per week to combat unexpected barriers (e.g., teacher strikes) (Wang et al., 2016). One trial reported that post-implementation, counselors who continued delivering TF-CBT in usual care changed the session frequency from weekly to two sessions per week to better accommodate their day-to-day roles (Dorsey et al. 2020a, b). Session length ranged from 30 to 120 min, and the number of TF-CBT sessions ranged from 8 to 32, with most (80%) trials aiming for the standard 12–16 sessions. One trial implemented an abbreviated, 8-session version that was tested in a pilot study given the implementation and scale-up advantages of brief treatments (Dorsey et al. 2020a, b). Two trials (20%) described recommendations for contextual modifications based on their findings. Lay counselors in one trial recommended shortening session length (Murray et al. 2013a, b). During this trial, TF-CBT was intended to be delivered in 60 min sessions but ranged from 30 to 120 min. Participants in another trial recommended adding counselor follow-up after completion of TF-CBT (Dorsey et al. 2020a, b), although it was not clear if this recommendation is specific to task-shifting or generalizable.

#### Content Modifications

The core components of TF-CBT follow the acronym PRACTICE: Psychoeducation and Parenting skills, Relaxation skills, Affective regulation skills, Cognitive coping skills, Trauma narration and processing, In vivo mastery of trauma reminders, Conjoint parent-child sessions, and Enhancing safety (Cohen et al., 2017). Three trials (30%) reported content modifications, all of which were surface-level language modifications that did not change core components of TF-CBT. For instance, one trial replaced technical language with more understandable terms (e.g., referring to sessions as “class” rather than therapy) (Dorsey et al. 2020a, b). This trial also offered recommendations for content modifications, including embedding procedures for safety planning and resources to link participants to services for other psychosocial needs within the intervention (Dorsey et al. 2020a, b). In another trial, lay counselors crafted the introduction to TF-CBT that was presented to clients at the beginning of treatment (Murray et al. 2013a, b).

### What Implementation Strategies are Used to Support Lay Counselor Delivery of TF-CBT?

Implementation strategies are activities aimed at facilitating successful implementation of TF-CBT by lay counselors. Specifically, we describe implementation strategies across the following categories: lay counselor training and supervision, TF-CBT fidelity monitoring, and other implementation strategies.

#### Training

All trials reported that lay counselors received training in TF-CBT (see Table 4 for training details). In nine trials (90%), lay counselors attended TF-CBT training sessions prior to implementation. The frequency and duration of training sessions varied, including two days, three days, 5–6 days, and 10 days of training. Most trials (*n* = 6; 60%) modified training by implementing ongoing training activities beyond completion of the initial sessions. These activities included a post-training period of practicing TF-CBT delivery with feedback, booster training sessions midway through implementation, and a post-training assessment. Similarly, one trial lengthened training for future initiatives based on feedback from lay counselors that the initial training (two, five-day sessions) was too condensed (Murray et al. 2013a, b). One trial reported that booster training sessions were also useful in training replacement counselors, which was common due to high counselor turnover (Dorsey et al. 2020a, b). Another modification to the structure of training was involving local trainers as lead trainers rather than intervention experts (*n* = 3; 30%). Local trainers were lay counselors who had experience in delivering TF-CBT in prior studies (Dorsey et al. 2020a, b) or leadership from the local organization who were involved in TF-CBT tailoring (Wang et al., 2016). In these trials, local trainers completed their own training, including a five-day Train-the-Trainer led by an experienced TF-CBT trainer or workshops that covered didactic techniques and demonstration of their teaching skills.

The content of lay counselor trainings retained standard components of TF-CBT training (e.g., psychoeducation on trauma and PTSD, didactic instruction on intervention strategies, role-play and practice groups, and manual review; Cohen et al., 2017). Modifications to training activities included adding components or modifying language of training materials. Following the Apprenticeship model (*n* = 5; 50%), intervention tailoring was included as a component of training. Lay counselors discussed and piloted ways to enhance fit of TF-CBT with the local setting, and modifications were reviewed by trainers to check adherence to fidelity. Similarly, another study described training as a “dialectical process,” wherein counselors provided feedback on material as it was taught (Wang et al., 2016). Three studies (30%) also added training on basic counseling skills (Li et al., 2023; O’Donnell et al., 2014; Wang et al., 2016), although details on these activities were not reported. In terms of language modifications, two studies replaced technical terms with plain language. In one trial, the lead trainer tailored existing materials prior to lay counselor training and presented modifications to TF-CBT and local experts to ensure that they were both adherent and understandable (e.g., changed “affective modulation” to “talking about feelings”). Another trial described intervention fidelity to counselors as “following the recipe,” and encouraged modifications that “spice it up” to fit the local context (Dorsey et al. 2020a, b).

#### Supervision

In all trials, lay counselors received ongoing supervision throughout TF-CBT delivery. Supervision details are presented in Table 4. Of the eight trials (80%) that reported frequency, most (*n* = 7) implemented weekly supervision, and one implemented pre- and post-session meetings. Three trials (30%) used a combination of in-person and virtual modalities, and one implemented virtual-only supervision. Virtual supervision took place via skype/phone call, texting support (e.g., SMS/WhatsApp), or listening to an audio or video role play. One trial described a plan to modify the frequency and modality of supervision in the intervention sustainment phase, during which supervision would occur less frequently (once per month) and less often in-person (Dorsey et al. 2020a, b). This trial also developed implementation guidelines and conducted educational outreach visits with lay counselors beforehand to enhance success of the virtual modality. In six trials (60%), supervision was led by local supervisors rather than external TF-CBT experts. Local supervisors were the local trainers described above (Dorsey et al. 2020a, b; Wang et al. 2016), or lay counselors who demonstrated strong skill uptake and leadership during training or in a prior study (Murray et al. 2013a, b, 2015). In all these trials, local supervisors received their own training and supervision, including regular virtual meetings with TF-CBT experts, local leadership, and/or completing workshops in supervision skills and models of clinical supervision. Lay counselor supervision included review of intervention fidelity, discussion of cultural modifications, case presentation, session planning, and addressing logistical barriers. During their supervision, local supervisors presented cases from lay counselor supervision to TF-CBT experts to ensure intervention fidelity. One trial reported that as local supervisors gained experience, the focus of their supervision shifted towards monitoring participant safety and logistics (Dorsey et al. 2020a, b).

#### Fidelity Monitoring

Most trials (*n* = 8; 80%) described procedures for fidelity monitoring to ensure counselor adherence to TF-CBT. Fidelity assessments included self-report checklists, detailed counselor-completed session notes, review of session audio/video recordings, observation of sessions by local supervisors or researchers, and/or local supervisor meetings with TF-CBT experts. See Table 4 for additional detail on fidelity monitoring procedures.

#### Other Implementation Strategies

Other implementation strategies were broadly defined as any activities beyond training and supervision that supported lay counselor delivery of TF-CBT. Some strategies focused on enhancing feasibility and resolving implementation barriers. For instance, two trials (20%) developed simplified step-by-step guidelines of session tasks to aid counselors in adhering to core components of fidelity (Dorsey et al. 2020a, b; Li et al. 2023). One trial described developing an “implementation coaching” plan (i.e., external facilitation in implementation science; Stetler et al., 2006), wherein local leaders and lay counselors met to identify anticipated implementation barriers and proactively generate solutions (Dorsey et al. 2020a, b). Three trials (30%) reported that because lay counselors often had roles and jobs outside of TF-CBT delivery, their competing responsibilities posed challenges to implementation (Dorsey et al. 2020a, b; Murray et al. 2013a, b, 2015). To address this, one trial described the need for monetary incentives for counselor time (Dorsey et al. 2020a, b) and two trials increased time dedicated to TF-CBT delivery (Dorsey et al. 2020a, b; Murray et al. 2013a, b). Collaborating with and fostering buy-in from leadership in the local setting with decision-making power over counselor schedules was critical for successful implementation. Collaboration included inviting local leadership to the TF-CBT training to enhance their knowledge of the program and facilitate buy-in (Dorsey et al. 2020a, b), disseminating a training program tailored to the local organization that included TF-CBT materials and a summary of its evidence base (Wang et al., 2016), and obtaining support from schools to facilitate recruitment and implementation (Li et al., 2023).

Another category of implementation strategies focused on lay counselor wellbeing. One trial provided counselors with a list of resources and protocol to refer clients for non-mental health related needs that emerged during treatment (Dorsey et al. 2020a, b). This effectively helped counselors manage the stress of learning about hardships faced by the families that was outside of the scope of TF-CBT. One trial outlined strategies that were specifically aimed at enhancing counselor confidence (e.g., encouragement, rewards) (Dorsey et al. 2020a, b), and another trial developed a detailed safety protocol to promote provider self-efficacy when conducting risk assessments (Murray et al. 2013a, b).

## Discussion

This review provides the first synthesis of modifications and strategies to support TF-CBT for delivery by lay counselors. In total, 10 trials were located that reported components of the modification process, intervention modifications, or implementation strategies (e.g., training and supervision) to support lay counselor delivered-TF-CBT. Most trials (90%) took place in LMICs, and all trials implemented culturally tailored TF-CBT in non-specialty mental health settings. The location, participant sample, and lay counselor characteristics varied across trials. The process of tailoring TF-CBT commonly consisted of multiphasic collaborations between researchers and local experts, sometimes spanning multiple research initiatives. All modification activities involved engagement of community members in the identification and operationalization of modifications. Modifications to TF-CBT were contextual (e.g., multiple sessions per week) or surface-level content modifications (e.g., language tailoring). Notably, no adaptations to core components of TF-CBT were reported. Training and supervision were modified to reduce involvement of external experts (e.g., reliance on local trainers) and to facilitate an ongoing learning process for lay counselors (e.g., post-training periods, booster sessions). Finally, additional implementation strategies targeted feasibility and logistical barriers (e.g., workload adjustments, fostering buy-in from community partners), and lay counselor wellbeing. Consistent with prior research testing the effectiveness of lay counselor-delivered treatments (Barnett, Lau et al., 2018; Connolly et al., 2021), TF-CBT was reported to be feasible and effective when delivered by lay counselors. When access to training and implementation supports are available, it appears that lay personnel can successfully deliver TF-CBT without prior specialized mental health training. Although prior reviews have demonstrated the effectiveness of task-shifted delivery of TF-CBT (Barnett, Lau et al., 2018; Connolly et al., 2021), none have synthesized modifications that are needed to inform future research and implementation efforts. This review fills this gap as the first aimed at characterizing strategies to facilitate dissemination of lay counselor-delivered TF-CBT, with potential utility that may extend beyond LMICs.

The process is the “how” of intervention tailoring and is critical in translating research to practice by providing a blueprint for future initiatives. Our review answers calls in the field for enhanced transparency and reporting of the modification process (Wiltsey Stirman et al., 2019) by describing strategies that researchers have employed to identify and operationalize modifications for lay counselor-delivered TF-CBT. Although specific frameworks (e.g., DIME, Apprenticeship model), activities (e.g., focus groups, interviews), and timepoints (e.g., pre-, during, and post-implementation) of modifications varied, a ubiquitous component of the process was community engagement. Many studies anchored the process of tailoring TF-CBT in community-based participatory research (CBPR) approaches to ensure that adaptations and implementation centered the needs and voices of those being served. Consistent with CBPR, community members (e.g., lay counselors, local leadership) guided adaptations pre-, during, and post-implementation through focus groups (Dorsey et al. 2020a, b; Li et al. 2023; O’Donnell et al. 2014) interviews (Murray et al. 2013a, b), and feedback during training and implementation (Dorsey et al. 2020a, b; Murray et al. 2013a, b, 2015; O’Callaghan et al. 2013; Wang et al. 2016). Because evidence-based interventions were developed and tested in controlled research environments that may differ from real-world settings, CBPR approaches can help ensure the effectiveness and sustainability of such interventions (Collins et al., 2018; Wallerstein & Duran, 2006). Findings from this review suggest that by empowering community members as equal partners in research and experts of their context, researchers built trusting community-researcher partnerships that laid the foundation for implementation, refinement, and scale-up of TF-CBT.

We also examined if components of TF-CBT warrant modification to enhance feasibility and sustainability of lay counselor delivery. This advances the literature on modifications to TF-CBT and task-shifting, which have previously focused on cultural adaptations (Mabunda et al., 2022; Metzger et al., 2021), toward those specific to the sustainment of task-shifting models. The absence of core content adaptations is an important finding given that intervention adaptation was a goal in many studies. This suggests that TF-CBT is overall acceptable, appropriate, and feasible for delivery by lay counselors, and that its core components can be preserved in task-shifting models. All modifications to TF-CBT were either contextual, to enhance fit with the local setting, or surface-level content modifications, highlighting that the active ingredients of TF-CBT should be preserved and only peripheral components refined to enhance success of task-shifting. Contextual modifications were most common and included embedding TF-CBT in existing lay counselor schedules (e.g., during 1-hour school breaks), implementing an abbreviated 8-session version, delivering multiple sessions per week, and allowing flexibility in session duration (30–120 min). TF-CBT typically includes approximately 12–16, 60-minute individual weekly sessions (Cohen et al., 2017), yet most studies did not fully adhere to this structure. Researchers recognized that the standard format of TF-CBT may be incompatible with the competing roles and responsibilities of lay counselors, and tailored delivery to fit the local context while adhering to the core intervention components. This underscores the importance of adopting a “flexibility within fidelity” approach to EBT implementation (Cohen et al., 2008; Kendall & Beidas, 2007), particularly in task-shifting models that introduce unique implementation barriers (e.g., counselor jobs outside of mental health delivery). Researchers also offered recommendations for content modifications, including adding resources in TF-CBT to link clients to services for other ongoing stressors that commonly arose during treatment yet were outside of the scope of counselor roles and training. This recommendation reflects the unique clinical needs of populations served by task-shifting, and the importance of tailoring evidence-based mental health treatments to meet these needs. Task-shifting is a particularly useful strategy for increasing access to mental health services in low-resource communities, where workforce shortages are concentrated. In addition to high trauma-related mental health needs, clients in these settings are likely to experience co-occurring psychosocial stressors (e.g., housing, financial, and food insecurity) that undermine their wellbeing and affect their ability to engage in mental health services. Ensuring TF-CBT considers the holistic needs of clients is important in not only providing effective care, but also for preserving counselor wellbeing by ensuring counselors are equipped with resources to meet client needs.

Effective training and supervision are critical to successful implementation of evidence-based practice. Most modifications to TF-CBT training were strategies to enhance counselor learning and ensure their preparedness and competency in delivering TF-CBT. Training requirements for TF-CBT certification include completion of a 10-hour online asynchronous web-course (TF-CBTWeb), a live two-day TF-CBT training led by a certified trainer, and a series of consultation sessions to support intervention delivery (for more information on TF-CBT certification, visit https://tfcbt.org/). Of note, TF-CBT certification criteria also include holding at least a Master’s degree in a mental health discipline and being a licensed provider in the US or Canada. Therefore, lay counselors are not eligible for certification even if the other training requirements are met. No trials implemented the self-paced web-course as a training activity. The web course is intended for mental health professionals, and language barriers and/or limited technology access may have also limited utilization of this training resource. Of the nine studies that implemented initial TF-CBT training sessions, eight increased the number of days (up to 10), and one study received direct feedback from counselors that the initial training (two five-day sessions) was too condensed. The frequency of this training modification suggests that dividing the components of standard TF-CBT training to be delivered over a longer duration may be more digestible for lay counselors, in turn enhancing their learning and self-efficacy. Similarly, training was often an ongoing process with recurring activities throughout implementation (e.g., post-training practice, booster sessions mid-implementation). Many studies followed the Apprenticeship Model of training, which provides specific guidelines on ongoing training and supervision activities for lay counselors (Murray et al., 2011). These insights highlight the importance of ongoing training for lay counselors, with implications for advancing the quality of training in evidence-based treatments broadly. While initial training sessions are a necessary component of training to improve therapist knowledge, “one-off” trainings do not result in sustained behavioral change, even among mental health specialists (Beidas & Kendall, 2010; Frank et al., 2020; Herschell et al., 2010; Murray et al., 2011). As such, intervention uptake, client outcomes, and fidelity adherence improve when trainings are followed by ongoing support such as consultation (Frank et al., 2020). Consistent with these recommendations, findings from the present study suggest that recurring training activities, such as those outlined in TF-CBT (e.g., ongoing consultation) and the Apprenticeship Model (e.g., practice groups) may enhance uptake and sustainability of evidence-based treatments. Lastly, studies enhanced the content of training by teaching basic counseling skills prior to intervention-specific components; however, details of these components were not provided. This modification warrants further attention given its importance in bridging the inherent the gaps in knowledge of lay counselors, who do not have prior formal training in providing mental health services.

Modifications to training and supervision also targeted capacity building by equipping local personnel to serve as trainers and supervisors and using technology to reduce the resource burden of supervision. Despite the centrality of training and supervision to effective implementation of evidence-based interventions, the resource-intensiveness of ongoing provider supports pose challenges for sustainability, even in mental health care settings (Marques et al., 2016). Identifying strategies to build local capacity for training and supervision is necessary for intervention uptake. This may be particularly important in task-shifting because mental health delivery is not central to lay counselors’ day-to-day roles. Trials implemented Train-the-Trainer models to expand training capacity. Train-the-Trainer involves intervention experts and certified trainers training local personnel on both intervention content and training skills, who then serve as local trainers. This approach confers advantages for extending the reach and sustainability of evidence-based interventions by reducing the need for external involvement. There is growing evidence that Train-the-Trainer models are effective in increasing clinician knowledge, improving patient outcomes (Pearce et al., 2012), and that local trainers can facilitate learning as effectively as master trainers (Triplett et al., 2020). Consistent with this literature, findings from the present review suggest that not only can lay counselors be effectively trained to deliver TF-CBT, but with adequate support, they can also become trainers and supervisors themselves, thereby reducing the need for external involvement.

Our review elucidated multi-level implementation determinants and barriers to TF-CBT delivery that are particularly pronounced in task-shifting models and require additional solutions. For instance, the competing responsibilities of lay counselors could not fully be remediated through modifications to training, supervision, and TF-CBT. Organizational-level factors (e.g., lack of leadership support, lack of protected time) are salient determinants of implementation even in specialty mental health settings (Damschroder et al., 2009). These barriers are especially potent in task-shifting, because counselors hold other jobs and do not work in settings designed to support mental health treatment delivery. Consistent with existing recommendations (Powell et al., 2015), engaging gatekeepers of the local setting (i.e., those with decision-making authority) in research emerged as an important strategy to resolve organizational barriers. By collaborating with local leadership, researchers fostered community buy-in which enabled workload adjustments and increased counselor time allocated to TF-CBT delivery. Counselor wellbeing also emerged as an important consideration. The intensity of trauma therapy can have negative impacts on counselor wellbeing, and these impacts may be more severe for therapists with personal trauma histories (Pearlman & Mac Ian, 1995). Because lay counselors are often members of the communities they serve, they may be more susceptible to the negative impacts of trauma-focused work. Developing context-specific supports (e.g., safety protocol, resource list for psychosocial stressors) and ensuring open communication with lay counselors (e.g., during supervision) may be important to facilitate their own coping and processing of trauma-focused work.

Findings from this systematic review can support dissemination of lay counselor-delivered TF-CBT, with implications for expanding access to trauma-focused care for youth globally. Investigators have successfully adopted task-shifting models to increase access to TF-CBT in LMIC’s where there are significant workforce shortages. No studies were found that implemented lay counselor-delivered TF-CBT in under-resourced settings in the US, despite rates of access to mental health care that are comparable to LMICs (Derr, 2016). Task-shifting directly addresses factors that maintain the treatment gap in the US, including the mental health workforce shortage and mental health stigma and medical mistrust among minoritized communities. There is some evidence for the effectiveness and acceptability of lay counselor treatment models in the US (e.g., peer-delivery, community health workers; Kanzler et al., 2024), but these have not been applied to TF-CBT. Given the success of lay counselor-delivered TF-CBT globally and its promise in addressing barriers to care in the US, extending this model to the US warrants consideration as a strategy to close trauma-related health disparities. Findings from our review offer strategies that can be applied to implement lay counselor-delivered TF-CBT in the US. These include establishing partnerships with trusted community organizations where youth and caregivers seek care (e.g., religious organizations, schools), tailoring TF-CBT for delivery in these settings, and training local personnel. Although this is a promising avenue to close the trauma-focused treatment gap, future research testing lay counselor-delivered TF-CBT in the US is needed to determine the utility of this model in higher-income countries, and if the strategies from global health researchers are generalizable to such settings. Nonetheless, by synthesizing strategies employed by global health researchers to support implementation of lay counselor-delivered TF-CBT, this study provides a blueprint to support scale-up of this treatment model globally.

### Limitations

This review provides the first synthesis of modifications to TF-CBT for lay counselor delivery. We note several limitations of this work. First, only studies published in English were included in this review due to resource constraints and language proficiency of the research team, which may introduce biases in the representation of evidence included. Second, because we aimed to synthesize modifications, we are unable to draw conclusions about the efficacy of specific strategies. Future research should examine the relative importance of modifications and implementation strategies to better understand which are essential for lay counselor-delivery. The lack of available demographic information (e.g., race/ethnicity) of clients and lay counselors is a notable limitation with implications for the generalizability of findings. Enhanced reporting of demographic characteristics is important to better tailor treatment delivery and implementation strategies to fit the local context and unique community needs. Although we sought to differentiate the process, treatment modifications, and implementation strategies, these categories may not reflect fully disparate constructs (e.g., the Apprenticeship Model is both an aspect of the modification process and implementation strategies), and there may be overlap in findings reported across sections. The details of modifications varied widely across studies and were not systematically reported, which further limited this review. In particular, modification processes and implementation strategies were often not clearly described, which aligns with findings from prior reviews that these activities are not consistently reported (Barnett et al. 2018a, b), posing challenges for comprehensively synthesizing these in-text. It is possible that some modification activities were omitted from papers due to various reasons (e.g., were not the main aim of the paper, due to page limits of journals), and as a result, would be missing from the present review. Additionally, this review was focused on characterizing adaptations that were relevant to task-shifting models and did not include cultural adaptations. All studies included in the review implemented culturally modified versions of TF-CBT and in some cases, it was difficult to discern whether the process and modifications reported were aimed at enhancing cultural relevance of the intervention or fit with the lay counselor treatment model. We attempted to resolve this through double-coding and consensus meetings during data extraction, but it is possible that relevant modifications were not included in this review if they were described by authors as cultural adaptations. These limitations underscore the importance of systematic reporting of key details of modifications (e.g., the purpose of each modification). Consistent with calls in the field, we recommend that researchers anchor reporting of modifications in established frameworks that have been developed to enhance consistency, such as FRAME (Wiltsey Stirman et al., 2019).

## Conclusion

Given the known health consequences of childhood trauma, strategies to close the mental health treatment gap and increase access to evidence-based interventions such as TF-CBT are urgently needed. The current review sought to synthesize how TF-CBT has been modified for delivery by lay counselors. Trials reported activities from the modification process, modifications made to TF-CBT, and implementation strategies (e.g., training and supervision) to support lay counselor delivery. Findings provide critical insights on how researchers and clinicians, both internationally and within the US, may apply task-shifting to increase access to TF-CBT in usual care settings.

**Table 4:** Training and supervision characteristics across trials of lay counselor-delivered TF-CBT

Trial	Training	Supervision	Fidelity Monitoring
1. Dorsey et al. (2019) Dorsey et al. (2020a, b) Dorsey et al., (2023) Johnson et al. (2024) Martin (2022) Meza et al. (2020) Triplett et al. (2021) Triplett et al. (2023a, b) Triplett et al. (2023a, b)	In person (5–6 days) training led by local trainer followed by practice delivering TF-CBT (2–3 weeks) (Apprenticeship model). Local trainers: Train-the-Trainer led by TF-CBT expert followed practice (2 months).	Weekly in-person or virtual (e.g., phone call, text) meetings with local supervisors. Local supervisors: weekly virtual consultation with TF-CBT experts (1–1.5 h).	Self-report fidelity checklist and supervisor observation of sessions.
2. Dorsey et al., (2022) Dorsey et al. (2020a, b) Woods-Jaeger et al. (2017)	In-person (10 day) training led by local trainers followed by practice delivering TF-CBT and booster training sessions (Apprenticeship model). Local trainers: Train-the-Trainer (5 days) led by experienced TF-CBT trainer.	Weekly in-person or virtual (skype/telephone) meetings with local supervisors. Local supervisors: weekly consultation calls with TF-CBT experts.	Counselor reports, session audio recordings, discussion.
3. Murray et al. (2013a, b) Murray et al. (2014)	Two live trainings (5 days each) led by lead author followed by practice (Apprenticeship model).	Group supervision (2 h/week) with local supervisors. Local supervisors: weekly consultation calls with TF-CBT experts.	Counselor-completed case notes of components delivered, logistics, details on implementation strategy.
4. Li et al. (2023)	Group training (3 days) led by three manual developers from local institution followed by test of knowledge/skills, booster training (1.5 days) in the midterm, detailed intervention manual.	Weekly virtual supervision from three manual developers.	Video-recorded therapy sessions and review of counselor reports.
5. Murray et al. (2013a, b)	In-person training followed by practice groups (Apprenticeship model).	Weekly (2–4 h/week) meetings with local supervisors. Local supervisors: weekly consultation calls with trainers (2 h/week).	Self-report fidelity checklist
6. Murray et al. (2015)	On-site training (10 days) led by TF-CBT experts (Apprenticeship model).	Weekly group meetings with local supervisors. Local supervisors: weekly consultation with TF-CBT experts.	Counselor-completed session notes reviewed by supervisors and TF-CBT experts during supervisor supervision.
7. O’Callaghan et al. (2013)	Facilitators studied the manualized intervention before each session.	Pre- and post-session meetings with the lead authors.	Lead researcher monitored each session.
8. O’Callaghan et al. (2015)	Training sessions (six total) on how to deliver the intervention, received intervention manuals.	Counselors received prior “in-the-field” supervision while delivering the intervention.	NR
9. O’Donnell et al. (2014)	In-person (10 day) training led by TF-CBT trainer followed by 1-month practice with expert oversight.	Weekly calls with US investigators and four in-person meetings.	Self-report fidelity checklists reviewed by US investigators.
10. Wang et al. (2016)	Two-day workshop (Day 1 basic helping skills, Day 2 TF-CBT and modifications) led by local staff who were involved in cultural adaptation and completed their own training.	Meetings with local supervisors. Local supervisors: completed workshops on supervision models, attend supervision with the organization director.	NR

*NR* not reported, *TF*-*CBT* trauma-focused cognitive behavioral therapy

## Supplementary Information

Below is the link to the electronic supplementary material.

Supplementary Material 1 (DOCX 30.4 KB)

## Data Availability

The data from this review are not publicly available but may be made available upon request. This systematic review was pre-registered on PROSPERO (CRD#).
