# Supplementary material for: Lay Counselor Delivery of Trauma-Focused Cognitive Behavioral Therapy (TF-CBT): A Systematic Review
Source: J Child Adolesc Trauma. 2026 Mar 26;19(3):1645–63. doi: 10.1007/s40653-026-00852-z (PMC13546183; doi:10.1007/s40653-026-00852-z)
Supplement: Supplementary file 1 [file 40653_2026_852_MOESM1_ESM.docx]

| **Supplementary Table 1**  *Data Extraction Form* *for Systematic Review of Lay Counselor Delivered TF-CBT* | |
| --- | --- |
| **Code** | **Reviewer Instructions** |
| Author, Year | Enter first author last name, year of publication. |
| Publication Type | Enter information on the type of article:   - Peer-reviewed research article - Discussion article - Conference abstract - Dissertation |
| Study Design | Select from the following:   - Quantitative - Qualitative - Mixed methods - Non-empirical |
| ***Details below pertain to the delivery/intended delivery of TF-CBT*** | |
| Country/Location | Enter the country where TF-CBT was (or is intended) to be implemented, and other specific details on location if provided. |
| Delivery Setting | Enter the setting in which TF-CBT was (or is intended) to be delivered. *Examples: school, community organization* |
| Patient population characteristics | Characteristics of the population whom TF-CBT is (or is intended) to be delivered to (i.e., youth). Include the following:   - Age - Race/ethnicity - Sample size - Other demographic information |
| Lay counselor type | State the type of persons who are considered “lay counselors” in the study.  *Examples: community volunteers, educators* |
| Lay counselor recruitment | State how lay counselors were identified and recruited (e.g., from community partnerships, public advertising). Include direct excerpts from manuscript when possible. |
| Lay counselor characteristics | Characteristics of lay counselors who delivered (or will be delivering) TF-CBT. Include the following details:   - Number of lay counselors - Age - Race/ethnicity - Education Level - Required/desired qualifications - Prior training in mental health/psychotherapy - Other demographic information |
| Version of TF-CBT | Enter the name of the version of TF-CBT that was implemented. For example, original TF-CBT, abbreviated TF-CBT, or name of culturally tailored intervention. |
| Culturally tailored version of TF-CBT? | Yes/No. |
| Intervention delivery details (format, modality, number of sessions, session length, frequency) | Enter details regarding how TF-CBT was/will be delivered. Include the following:   - Format (e.g., group, individual) - Modality (in-person, virtual) - Number of sessions - Session duration - Session frequency |
| Outcomes | List all main outcomes reported in the paper. E.g., effectiveness, feasibility, acceptability, appropriateness, fidelity. |
| Conclusion | State the conclusion pertaining to the outcome type based on findings. |
| ***Main Review Research Questions: Extracting Content for Narrative Synthesis.*** For each item below, if “Y,” paste the relevant text excerpts into the following column. The text excerpts will be used for subsequent narrative synthesis. | |
| Describes modification process? | Y/N; *how the need for adaptation was determined, how adaptations were operationalized and tested. Information relevant to the process of adapting rather than adaptations themselves.* |
| Describes training (including any modifications to content/structure of training)? | Y/N; *how lay counselors were trained in TF-CBT* |
| Describes supervision (including any modifications)? | Y/N; *supervision that lay counselors received throughout TF-CBT delivery* |
| Describes fidelity monitoring (including any modifications)? | Y/N; *procedures used to monitor fidelity adherence* |
| Describes modifications to TF-CBT? | Y/N; *changes made to TF-CBT content or how it was delivered to support task shifting model*  *Note: these are only adaptations related to lay counselor delivery. If cultural adaptations specific to the population rather than related to the task shifting model are described and no other adaptations, this should be “N”* |
| Describes other implementation strategies (including modifications)? | Y/N. *Implementation strategies are defined as: “methods or techniques used to enhance the adoption, implementation, and sustainability of a clinical program or practice.” Implementation strategies cover a wide range of activities across multiple levels and systems involved in implementation.* [*ERIC*](https://implementationscience.biomedcentral.com/articles/10.1186/s13012-015-0209-1) *provides a comprehensive list of examples. Refer to this list to determine if activities may be considered an implementation strategy.* |
| Describes barriers to lay counselor delivery? | Y/N |
| Describes recommendations for future modifications to support lay counselor delivery? | Y/N |

| **Supplementary Table 2.**  *Risk of Bias and Quality Assessment Scores Across Included Trials of Lay Counselor Delivered TF-CBT Based on QuADS* | | | | | | | | | | | | | |
| --- | --- | --- | --- | --- | --- | --- | --- | --- | --- | --- | --- | --- | --- |
|  | QuADS Criteria | | | | | | | | | | | | |
| Article (Author, Year) | 1 | 2 | 3 | 4 | 5 | 6 | 7 | 8 | 9 | 10 | 11 | 12 | 13 |
| Dorsey et al., (2019) | 3 | 3 | 3 | 3 | 3 | 3 | 3 | 3 | 2 | 3 | 3 | 3 | 2 |
| Dorsey, Gray et al. (2020) | 3 | 3 | 3 | 3 | 3 | 3 | 3 | 3 | N/A | 3 | 3 | 3 | 3 |
| Dorsey, Lucid et al. (2020) | 3 | 3 | 3 | 3 | 3 | 3 | 3 | 3 | 3 | 3 | 3 | 3 | 3 |
| Dorsey et al., (2022) | 3 | 3 | 3 | 3 | 3 | 3 | 3 | 3 | 3 | 3 | 3 | 2 | 3 |
| Dorsey et al., (2023) | 2 | 3 | 2 | N/A | N/A | N/A | N/A | N/A | N/A | N/A | N/A | 3 | 2 |
| Johnson et al., (2024) | 3 | 3 | 3 | 3 | 3 | 2 | 3 | 3 | 3 | 3 | 3 | 3 | 3 |
| Li et al., (2023) | 2 | 3 | 3 | 3 | 3 | 3 | 3 | 3 | 3 | 2 | 3 | 2 | 2 |
| Martin et al., (2022) | 3 | 3 | 3 | 3 | 3 | 3 | 3 | 3 | 3 | 3 | 3 | 3 | 3 |
| Meza et al., (2020) | 3 | 3 | 3 | 3 | 3 | 2 | 3 | 3 | 3 | 3 | 3 | 2 | 2 |
| Murray Familiar et al., (2013) | 2 | 3 | 3 | 3 | 3 | 3 | 3 | 3 | 3 | 2 | 3 | 3 | 2 |
| Murray, Dorsey et al., (2013) | 3 | 3 | 3 | 3 | 3 | 3 | 3 | 3 | 3 | 3 | 3 | 3 | 3 |
| Murray et al., (2014) | 3 | 3 | 3 | 3 | 3 | 2 | 3 | 3 | 3 | 3 | 3 | 2 | 3 |
| Murray et al., (2015) | 2 | 3 | 3 | 3 | 3 | 3 | 3 | 3 | 3 | 3 | 3 | 2 | 3 |
| O’Callaghan et al., (2013) | 2 | 3 | 3 | 3 | 3 | 3 | 3 | 3 | 3 | 3 | 3 | 2 | 3 |
| O’Callaghan et al., (2015) | 3 | 3 | 3 | 3 | 3 | 3 | 3 | 3 | 3 | 3 | 3 | 2 | 3 |
| O’Donnell et al., (2014) | 3 | 3 | 3 | 3 | 3 | 3 | 3 | 3 | 3 | 3 | 3 | 3 | 3 |
| Triplett et al., (2021) | 3 | 3 | 3 | 3 | 3 | 3 | 3 | 3 | N/A | 3 | 3 | 3 | 3 |
| Triplett, Johnson et al., (2023) | 3 | 3 | 3 | 3 | 3 | 3 | 3 | 3 | 3 | 3 | 3 | 3 | 3 |
| Triplett, Mbwayo et al., (2023) | 3 | 3 | 3 | 3 | 3 | 3 | 3 | 3 | 3 | 3 | 3 | 3 | 3 |
| Wang et al., (2016) | 3 | 3 | 3 | 3 | 3 | 3 | 3 | 3 | 3 | 3 | 3 | 2 | 3 |
| Woods-Jaeger et al., (2017) | 3 | 3 | 3 | 3 | 3 | 3 | 3 | 3 | 3 | 3 | 3 | 3 | 3 |
| **M (SD)** | 2.76 (.44) | 3 (0) | 2.95 (.22) | 3 (0) | 3 (0) | 2.85 (.37) | 3 (0) | 3 (0) | 2.94 (.24) | 2.90 (.31) | 3 (0) | 2.62 (.50) | 2.76 (.44) |
| *Note:* QuADS = Quality Assessment for Diverse Studies. Domains are scored on a 4-point scale from 0 (not at all) to 3 (complete). Domains include 1 = Theoretical or conceptual underpinning to the research, 2 = Statement of research aims, 3 = Clear description of research setting and target population, 4 = Study design is appropriate to address stated research aims, 5 = Appropriate sampling, 6 = Rationale for choice of data collection tools, 7 = Appropriate format and content of data collection tool, 8 = Description of data collection procedure, 9 = Recruitment data provided, 10 = Justification for analytic method selected, 11 = Appropriate analysis method, 12 = Stakeholder involvement, 13 = Strengths and limitations discussed. | | | | | | | | | | | | | |

| **Supplementary Table 3**  *List of Full-Text Articles Excluded from Review (N = 27) and Reasons for Exclusion* | |
| --- | --- |
| **Author (Year)** | **Reason for Exclusion** |
| Barnett et al., (2021) | Wrong publication type |
| Barnett et al., (2023) | Wrong publication type |
| Barnett, Gonzalez, et al. (2018) | Wrong publication type |
| Barnett, Lau, & Miranda, (2018) | Wrong publication type |
| Bass & Hamdani (2019) | Wrong intervention |
| Bentley et al., (2021) | Wrong intervention |
| Bosqui et al., (2024) | Wrong publication type |
| Brown et al., (2017) | Wrong publication type |
| Chaudhry et al., (2022) | Wrong intervention |
| Espeleta et al., (2022) | Wrong providers |
| Gone & Trimble (2012) | Wrong publication type |
| Hoagwood et al., (2010) | Wrong publication type |
| Holguin (2018) | Wrong intervention |
| Javadi et al., (2017) | Wrong publication type |
| Kohrt & Bhardwaj (2019) | Wrong intervention |
| Le et al., (2022) | Wrong publication type |
| Mabunda et al., (2022) | Wrong publication type |
| Madigan et al., (2015) | Wrong providers |
| Magidson et al., (2017) | Wrong intervention |
| Naeem (2019) | Wrong publication type |
| Pederson et al., (2020) | Wrong outcomes (Does not report modification/implementation of TF-CBT for lay counselor delivery) |
| Rose et al., (2022) | Wrong publication type |
| Shahmalak (2021) | Wrong publication type |
| Stadnick et al., (2020) | Wrong providers |
| Vally & Abrahams (2016) | Wrong publication type |
| Ventevogel & Spiegel (2010) | Wrong outcomes (Does not report modification/implementation of TF-CBT for lay counselor delivery) |
| Woody et al., (2015) | Wrong providers |
